# Pulmonary Fibrosis After COVID-19 Pneumonia

**DOI:** 10.7759/cureus.13923

**Published:** 2021-03-16

**Authors:** Bilal Malik, Basel Abdelazeem, Abhijeet Ghatol

**Affiliations:** 1 Internal Medicine, McLaren Health Care, Flint/Michigan State University, Flint, USA; 2 Pulmonary and Critical Care Medicine, McLaren Health Care, Flint, USA

**Keywords:** covid, covid-19, pulmonary critical care, pulmonary disease, medical icu, respiratory disease, fibrotic lung disease, pulmonary fibrosis

## Abstract

A novel coronavirus, severe acute respiratory syndrome coronavirus 2 (SARS-CoV-2), also known as coronavirus disease 2019 (COVID-19), emerged in Wuhan, China, and rapidly spread across the world. Today, we present an interesting case of a patient with no prior history of pulmonary disease who was diagnosed with COVID-19, recovered after a prolonged hospital course, and was diagnosed with pulmonary fibrosis requiring oxygen therapy thereafter. The patient is currently on pirfenidone and has had a significant improvement in his functional status. His oxygen requirements have decreased, and repeat computed tomography (CT) scanning has demonstrated improvement in the extent of his pulmonary fibrosis. This case highlights the possibility of pulmonary fibrosis being a major complication among COVID-19 survivors and the importance of using pirfenidone in the management of such cases.

## Introduction

Idiopathic pulmonary fibrosis (IPF), also called cryptogenic fibrosing alveolitis, is the most common form of idiopathic interstitial pneumonia [1]. IPF usually affects adults, with a predominance in men more often than women [2], and presents with chronic and progressive dyspnea associated with a dry cough. High-resolution computed tomography (CT) scanning with clinical correlation is satisfactory to make the diagnosis without the need for lung biopsy for histopathological confirmation [3,4]. Management includes smoking cessation, long-term oxygen therapy in patients with hypoxemia [5,6], anti-fibrotic medications such as pirfenidone or nintedanib [7], and lung transplant in patients who are suitable candidates [6].

To the best of our knowledge, only a limited number of cases of pulmonary fibrosis in coronavirus disease 2019 (COVID-19) survivors have been reported. We present the case of a 67-year-old male who was diagnosed with COVID-19, had an extensive intensive care unit (ICU) stay, developed pulmonary fibrosis after recovery, and was discharged with oxygen supplementation to maintain his oxygenation. This case report aims to raise awareness about pulmonary fibrosis as a complication of COVID-19, as well as early identification and initiation of management with oxygen, steroids, and anti-fibrotic therapy to maximize quality of life.

## Case presentation

**Figure 1:**
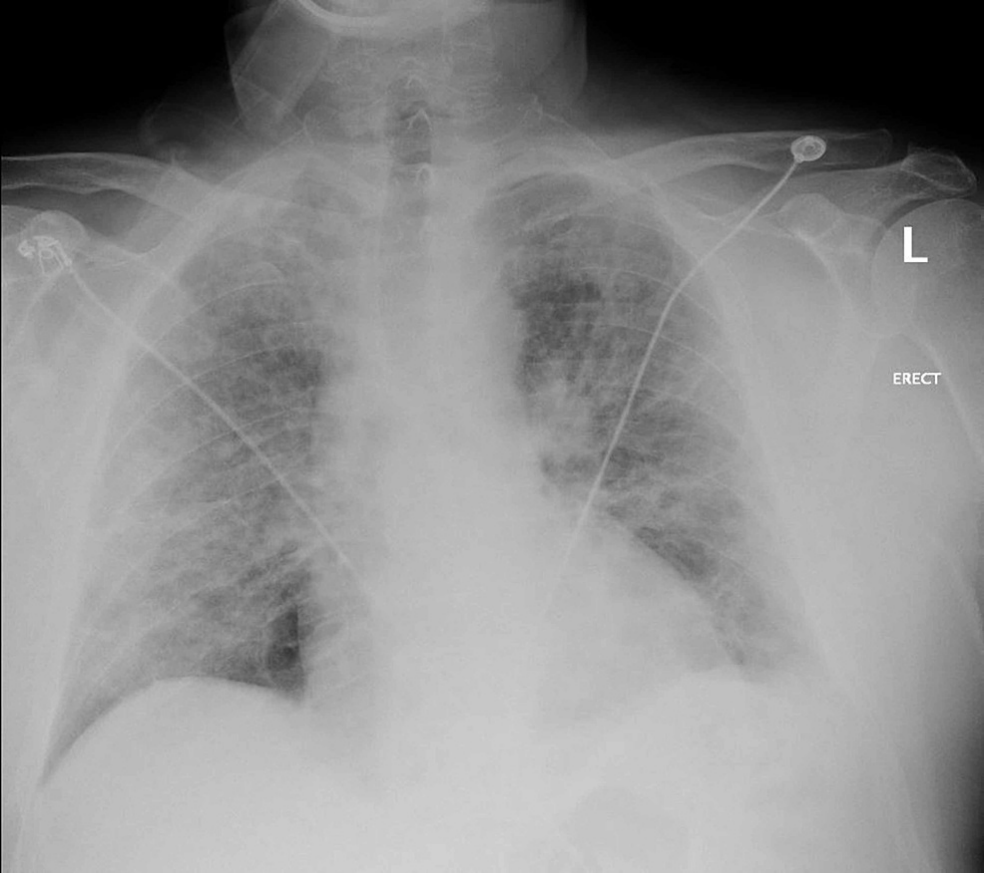
Chest X-ray on presentation showing diffuse patchy/interstitial opacities consistent with atypical infection No frank lobar consolidation is seen.

Our patient was a 67-year-old Caucasian male patient with a past medical history of hypertension and hyperlipidemia who presented with a five-day history of cough, shortness of breath, subjective fever, body aches, and diarrhea. The day prior to presentation, he was prescribed azithromycin by his primary care physician. This failed to improve his condition and prompted him to seek further care for progressive symptoms. Examination revealed a blood pressure of 137/80 mmHg, heart rate of 97 beats per minute, and oxygen saturation of 77% on room air with a respiratory rate of 24 breaths per minute. Breathing was labored with bilateral widespread crackles on auscultation. He complained of chest soreness and was febrile at 101°F. On interrogation, the patient denied any recent travel or known sick contacts. Chest X-ray on presentation demonstrated diffuse patchy interstitial opacities (Figure 1). EKG (electrocardiogram) did not demonstrate ST-segment changes. COVID-19 testing returned positive on admission day 2.

The patient was initially given a 5 liters per minute (lpm) nasal cannula in an attempt to maintain his oxygenation. His respiratory support was escalated to 100% fraction inspired oxygen (FiO_2_) high-flow nasal cannula with a flow rate of 60 lpm, alternating with non-invasive ventilation with bilevel at 16/6 and FiO_2_ between 80% and 100%. He was transferred to the ICU and commenced on methylprednisolone, ceftriaxone, which was later changed to piperacillin-tazobactam, azithromycin, and remdesivir. Blood and sputum cultures were negative. Laboratory data (Table 1) revealed mild leukocytosis (white blood cell [WBC] count) with lymphopenia and elevated inflammatory markers, such as C-reactive protein (CRP), troponins, lactate dehydrogenase (LDH), and ferritin. D-dimer levels were elevated and subsequently declined while on anticoagulation therapy.

**Table 1:** Inflammatory markers during ICU management CRP, C-reactive protein; WBC, white blood cell; LDH, lactate dehydrogenase; CK, creatine kinase

Lab values	Day 1	Day 12	Day 21	Day 30
D-dimer (mg/L)	1.43	0.92	0.41	0.33
CRP (mg/dL)	30.1	0.9	0.8	1.0
Troponins (ng/mL)	0.21	0.03	0.07	-
WBC count (x10^3^/uL)	14.23	19.02	16.70	14.64
Absolute lymphocytes (x10^3^/uL)	0.79	0.83	0.54	0.95
LDH (U/L)	260	310	296	399
Ferritin (ng/mL)	784	3204.5	1658.1	3120.6
CK (U/L)	50	18	56	53
Procalcitonin (ng/mL)	0.73	0.08	0.06	0.08

The patient was treated with nebulized breathing treatments, diuretics (furosemide), colchicine, intravenous heparin (which was later transitioned to apixaban), dexamethasone, amiodarone, a 7-day course of piperacillin-tazobactam, and remdesivir over the course of a month admission in the ICU. Over this period, he continued to require significant support with high-flow nasal cannula but was gradually weaned down to a normal nasal cannula.

**Figure 2:**
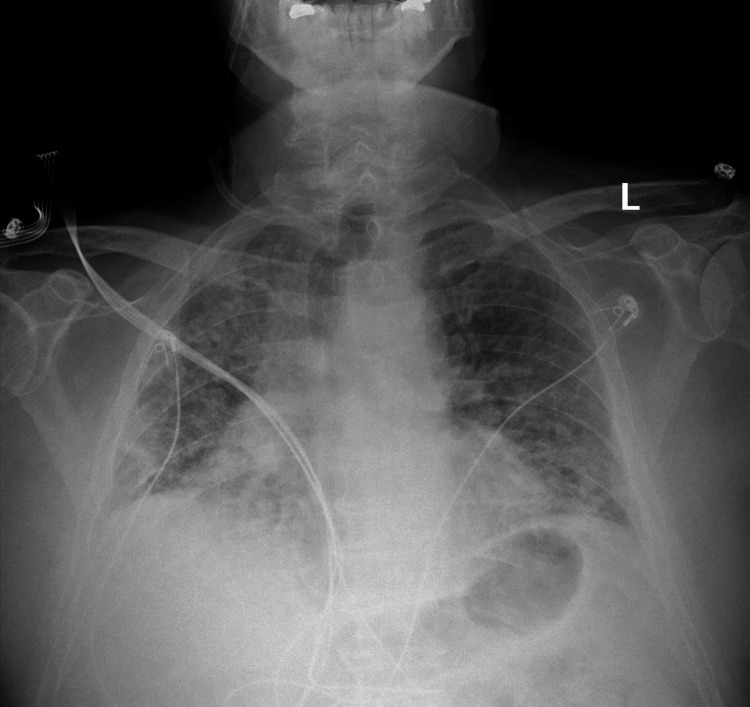
Chest X-ray at week six: diffuse bilateral pulmonic opacities, predominantly interstitial, are once again noted.

**Figure 3:**
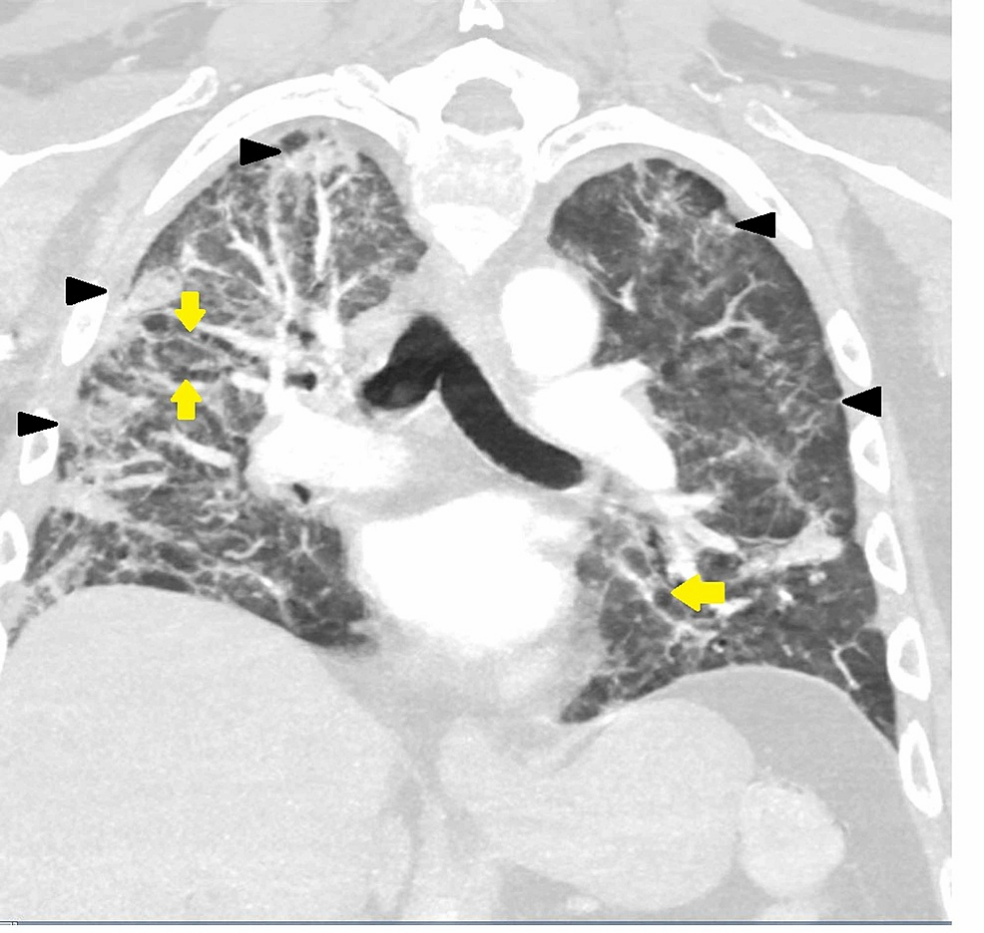
CT of the chest (coronal view) at week six: extensive diffuse bilateral pulmonary ground-glass opacities and air-space disease with architectural distortion bronchiectasis are seen. COVID-19 pneumonia/scarring is also noted. Black arrowheads show ground-glass opacities; yellow arrows show bronchiectasis

**Figure 4:**
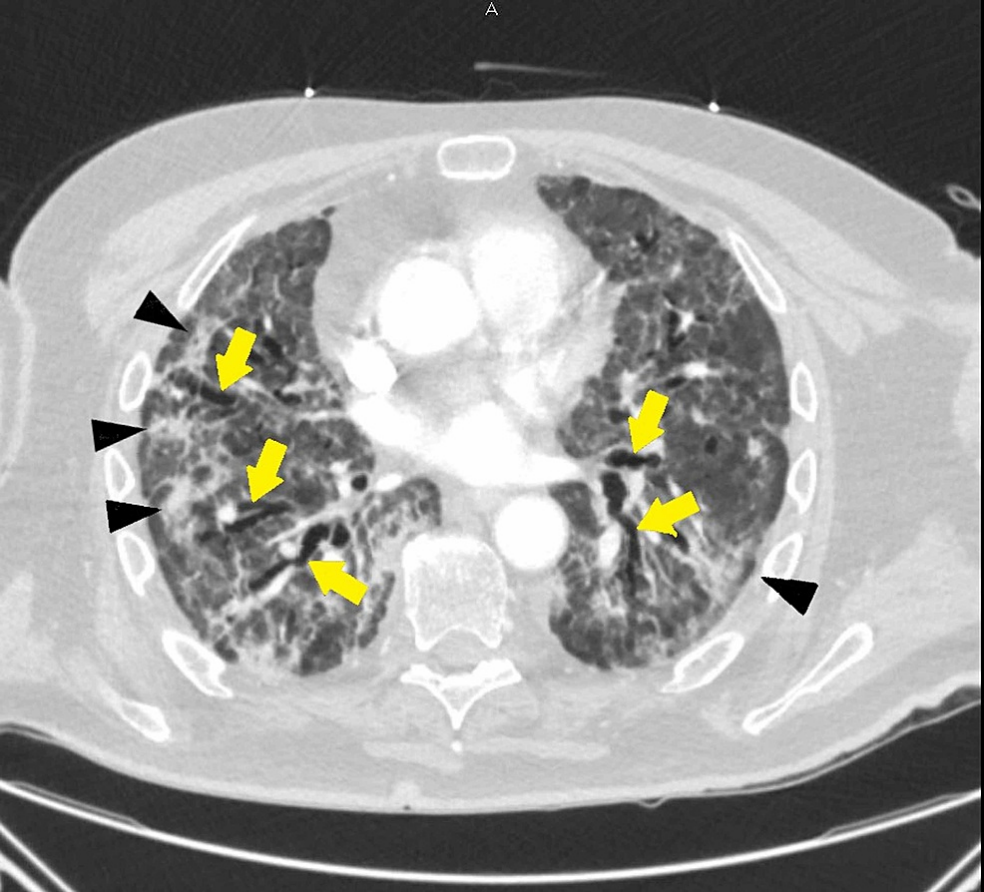
CT of the chest (transverse view) at week six: extensive diffuse bilateral pulmonary ground-glass opacities and air-space disease with architectural distortion bronchiectasis are seen. COVID-19 pneumonia/scarring is also noted. Black arrowheads show ground-glass opacities; yellow arrows show bronchiectasis

Once recovered from the acute illness, the patient continued to have shortness of breath requiring oxygen support to maintain his saturations. He was transferred to a long-term acute care hospital where he received physical therapy. Upon discharge, which was six weeks from the initial presentation, the chest X-ray revealed persistent diffuse bilateral pulmonic opacities (Figure 2), and a CT scan revealed pulmonary fibrosis (Figures 3, 4). He was maintained on a steroid dosage of 40 mg of oral prednisone per day for 10 weeks in view of the pulmonary fibrosis on imaging. He was then transitioned to 20 mg for four weeks. Currently, the patient is on 10 mg prednisone daily.

**Figure 5:**
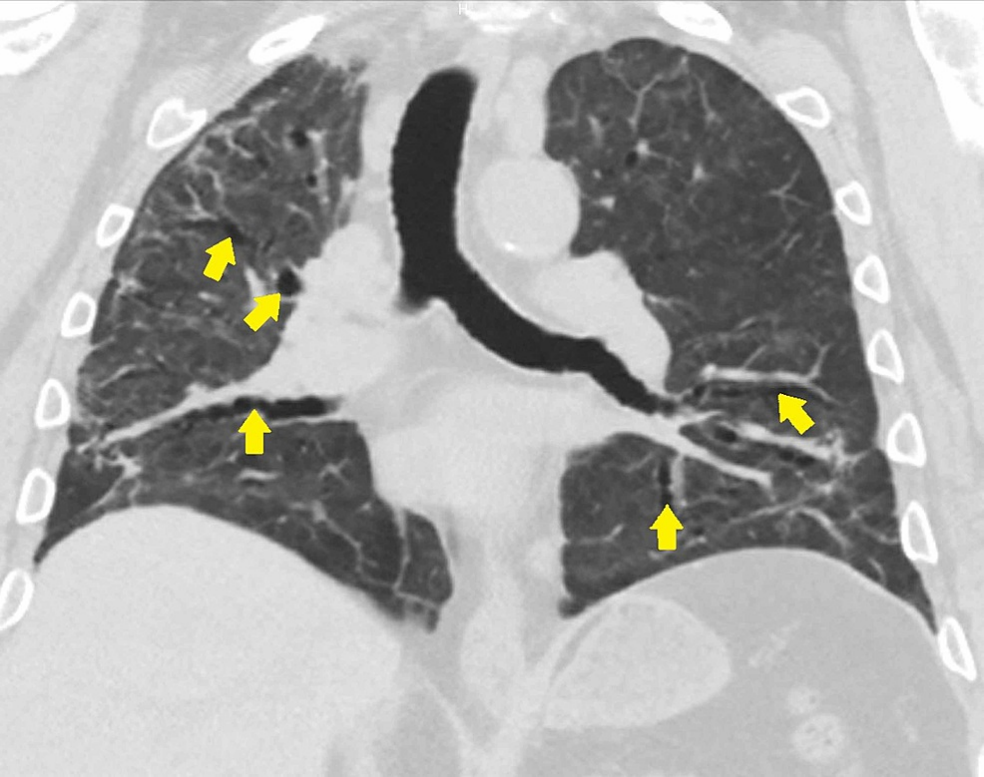
High-resolution CT of the chest (coronal view) at week 16: after five weeks of pirfenidone therapy, there is mild bronchiectasis. There is no reticular nodular density or significant ground-glass opacity. Yellow arrows show bronchiectasis

**Figure 6:**
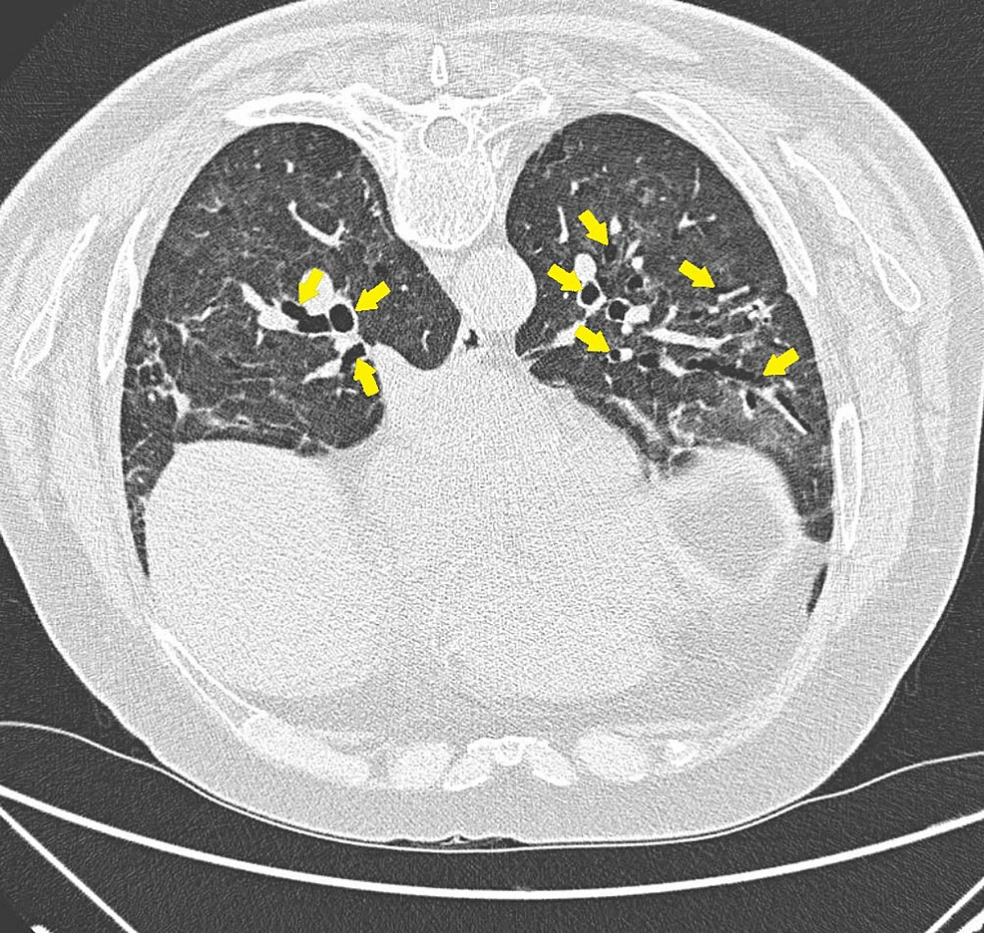
High-resolution CT of the chest (transverse view) at week 16: after five weeks of pirfenidone therapy, there is mild bronchiectasis. There is no reticular nodular density or significant ground-glass opacity. Yellow arrows show bronchiectasis

He was initiated on pirfenidone as an outpatient at 12 weeks from presentation. He is currently tolerating the full dose of pirfenidone at 801 mg three times per day with no adverse effects while concurrently taking 10 mg of prednisone daily. He has shown clinical improvement, as evidenced by a decreasing supplemental oxygen requirement from high-flow nasal cannula (100% at 60 lpm) and BiPAP ([bilevel positive airway pressure] 16/6) to 2 lpm nasal cannula, as well as improvement in the extent of his pulmonary fibrosis on serial CT scan of the chest (Figures 5, 6). The patient is currently still living and has had gradual improvements to his respiratory status since his discharge.

## Discussion

Pulmonary fibrosis is a pathologic sequela of unsuccessful reconstruction of damaged alveolar epithelium and persistent fibroblasts, causing excessive deposition of collagen and extracellular matrix components [8]. The acute and chronic inflammation causes sustained alveolar epithelial damage, which causes the overexpression of proinflammatory cytokines (TGF-β [transforming growth factor-beta], TNF-α [tumor necrosis factor-alpha], IL-6 [interleukin-6]), activating fibroblasts and myofibroblasts to cause excessive deposition of collagen in the extracellular matrix [9].

Our patient developed pulmonary fibrosis after recovering from an ICU-managed COVID-19 pneumonia. Steroids have become a vital component of the treatment for COVID-19 due to their efficacy in reducing the 28-day mortality in critically ill patients [10]. We propose that they may have a therapeutic role in treating pulmonary fibrosis. However, the dosage and duration need to be determined by longitudinal studies in the subset of COVID-19 patients developing pulmonary fibrosis.

Pirfenidone has a downregulating effect on cytokines as TGF-β1, connective tissue growth factor (CTGF), platelet-derived growth factor (PDGF), and TNF-α [3,11]. In addition to being a reactive oxygen species (ROS) scavenger, it also downregulates ACE receptor expression [4,12], one of the major receptors in COVID-19 pathogenesis. The role of pirfenidone is being studied in its efficacy for pulmonary fibrosis in COVID-19. Our patient received a 10-week course of 40 mg prednisone and subsequently four weeks of 20 mg prednisone. Currently, he is receiving 10 mg prednisone daily. The patient was started on pirfenidone as an outpatient at 12 weeks from presentation and has since had marked improvements in his respiratory symptoms and functional capacity, with a marked decrease in his oxygen requirements and significant improvement in the CT scans, as shown in Figures 5, 6.

To the best of our knowledge at this time, there is limited literature providing information, evidence, and recommendations for the management of pulmonary fibrosis in patients with previous COVID-19 infection. Table 2 summarizes the available case reports documenting fibrotic lung changes after COVID-19 infection. As more data emerge, directions for future research include analyzing novel therapeutic agents in this subset of pulmonary fibrosis patients, assessing specific pathophysiological mechanisms of pulmonary fibrosis in COVID-19 survivors, and examining the relationship between treatment modalities for initial COVID-19 infections and the development of pulmonary fibrosis as a subsequent complication.

**Table 2:** Summary of available case reports including patients with the development of fibrotic changes after COVID-19 CT, computed tomography; GERD, gastroesophagheal reflux disease; ECMO, extracorporeal membrane oxygenation; DLCO, diffusing capacity of the lung for carbon monoxide; FVC, forced vital capacity; FEV_1_, forced expiratory volume in the first second of the forceful exhalation; HRCT, high-resolution computed tomography

Study	Patient demographics, age (years) and gender	Comorbidities	Intervention	Respiratory support	CT findings/histology/supporting evidence of fibrosis
Okamori et al., 2020 [13]	60 M	Dyslipidemia, GERD	Levofloxacin, ciclesonide, corticosteroids, and favipiravir	Nasal cannula	CT scan revealed consolidation accompanied by reversed halo sign, traction bronchiectasis, and volume loss of the lower lobes
	61 F	Asthma, hypothyroidism, hypertension	Ceftriaxone, azithromycin, favipiravir, steroids, and hydroxychloroquine	Nasal cannula	CT scan demonstrated bilateral consolidations, some of which showed band-like shapes and distributed in the subpleural or peri-bronchial region, with traction bronchiectasis
Bharat et al., 2020 [14]	43 M	Diabetes type 2	Remdesivir, convalescent plasma, pathogen-directed antibiotics, as well as steroids	Mechanical ventilation and veno-venous ECMO	-
	28 F	Neuromyelitis optica	Broad-spectrum and pathogen-directed antibiotics, remdesivir, hydroxychloroquine, tocilizumab, and convalescent plasma	Mechanical ventilation and veno-venous ECMO	Histology: lung alveoli in the explanted lung from case 1 demonstrating hemorrhage, interstitial fibrosis, and prominent reactive pneumocytes
	62 M	Hypertension	Remdesivir, convalescent plasma, antibiotics, and dexamethasone	Veno-venous ECMO	Histology: bronchiolitis and bronchiolar fibrosis with microscopic honeycombing was observed for the explanted lung
Zha et al., 2021 [15]	68 M	Hypertension, diabetes type 2	Lopinavir-ritonavir	Mechanical ventilation with progression to tracheostomy	Pulmonary function test indicated restrictive lung function defect, with decreased FVC of predicted (62.3%) and DLCO of predicted (49.6%), but FEV_1_/FVC was at the normal range of 80.1%. Obvious architectural distortion, bronchial dilatation, and volume loss in bilateral lungs suggestive of fibrotic changes on chest CT.
Picchi et al., 2020 [16]	70 F	Light smoker 40 years before	Steroids, tocilizumab, azithromycin, lopinavir/ritonavir, hydroxychloroquine, enoxaparin	Nasal cannula	1-month follow-up CT scan showed disease progression with increasing range of ground-glass density patches and consolidation and scant fibrous interstitial stripes.
	88 F	Hypertension, diabetes type 2	Lopinavir/ritonavir, hydroxychloroquine, enoxaparin, steroids, levofloxacin, and ceftriaxone	High-flow oxygen	1-month follow-up CT scan showed decreasing range of ground-glass density patches and consolidation; new thin fibrous interstitial stripes appeared.
	63 F	Hypertension	Lopinavir/ritonavir, enoxaparin, steroids, azithromycin, ceftriaxone,	None	Illness day 18 CT scan revealed consolidation shadow in bilateral lung view, interlobular septal thickening with bronchiolectasis, and diffuse fibrotic evolution of the interstitial inflammation.
Tale et al., 2020 [17]	48 M	None	Low molecular weight heparin, dexamethasone, and antipyretics	Nasal cannula	HRCT of the chest was performed, which showed architectural distortion, interlobar septal thickening, and traction bronchiectasis features.

## Conclusions

Pulmonary fibrosis has been implicated as one of the complications of COVID-19. Currently, there is no effective treatment for pulmonary fibrosis, with lung transplantation being the only curative option. We presented a case of a 67-year-old Caucasian male patient who developed pulmonary fibrosis after COVID- 19 pneumonia and responded well to steroids and the anti-fibrotic agent pirfenidone. Further clinical trials are needed to assess appropriate dosing, duration, efficacy, and safety of novel anti-fibrotic agents in the management of COVID-19 related pulmonary fibrosis.
